# Assessment of Initial Depressive State and Pain Relief With Ketamine in Patients With Chronic Refractory Pain

**DOI:** 10.1001/jamanetworkopen.2023.14406

**Published:** 2023-05-19

**Authors:** Marion Voute, Céline Lambert, Bruno Pereira, Gisèle Pickering

**Affiliations:** 1 Centre Hospitalier Universitaire (CHU) Clermont-Ferrand, Institut National de la Santé et de la Recherche Médicale (INSERM) 1405, Centre d’Investigation Clinique 1405, Platform of Clinical Investigation, Clermont-Ferrand, France; 2 CHU Clermont-Ferrand, Direction de la Recherche Clinique et de l’Innovation, Biostatistics Unit, Clermont-Ferrand, France; 3 University Clermont Auvergne, INSERM 1107, Neuro-Dol, Clermont-Ferrand, France

## Abstract

**Importance:**

Repeated ketamine administration is common in treatment-refractory chronic pain, but ketamine analgesic and antidepressant effects are poorly understood in patients with chronic pain with depression symptoms.

**Objective:**

To determine clinical pain trajectories with repeated ketamine administrations, exploring whether ketamine dose and/or pretreatment depressive and/or anxiety symptoms may mediate pain relief.

**Design, Setting, and Participants:**

This nationwide, multicenter, prospective cohort study included patients in France with treatment-refractory chronic pain who received repeated ketamine administration, over 1 year, according to ketamine use in their pain clinic. Data were collected from July 7, 2016, through September 21, 2017. Linear mixed models for repeated data, trajectory analysis, and mediation analysis were performed from November 15 to December 31, 2022.

**Interventions:**

Ketamine administration in cumulative dose (milligrams) over 1 year.

**Main Outcomes and Measures:**

Primary outcome was mean pain intensity (0-10 on the Numerical Pain Rating Scale [NPRS]), assessed every month for 1 year by telephone, after inclusion in the hospital. Depression and anxiety (Hospital Anxiety and Depression Scale [HADS]), quality of life (12-item Short Form Health Survey [SF-12]), cumulative ketamine dose, adverse effects, and concomitant treatments were secondary outcomes.

**Results:**

A total of 329 patients (mean [SD] age, 51.4 [11.0] years; 249 women [75.7%] and 80 men [24.3%]) were enrolled. Repeated ketamine administration was associated with a decrease of NPRS (effect size = −0.52 [95% CI, −0.62 to −0.41]; *P* < .001) and an increase of SF-12 mental health (39.7 [10.9] to 42.2 [11.1]; *P* < .001) and physical health (28.5 [7.9] to 29.5 [9.2]; *P* = .02) dimension scores over 1 year. Adverse effects were in the normal range. There was a significant difference between patients without and with depressive symptoms in pain diminution (regression coefficient, −0.04 [95% CI, −0.06 to −0.01]; omnibus *P* = .002 for interaction of time × baseline depression [HADS score ≤7 or >7]). The mediation model showed that ketamine dose was not associated with pain diminution (*r* = 0.01; *P* = .61) and not correlated with depression (*r* = −0.06; *P* = .32), and that depression was associated with pain diminution (regression coefficient, 0.03 [95% CI, 0.01-0.04]; *P* < .001), whereas ketamine dose was not (regression coefficient, 0.00 [95% CI, −0.01 to 0.01]; *P* = .67). The proportion of reduction of pain mediated by baseline depression was 64.6%.

**Conclusions and Relevance:**

The findings of this cohort study on chronic refractory pain suggest that depression (and not ketamine dose or anxiety) was the mediator of the association of ketamine with pain diminution. This finding provides radically new insights on how ketamine reduces pain primarily by dampening depression. This reinforces the need for systematic holistic assessment of patients with chronic pain to diagnose severe depressive symptoms where ketamine would be a very valuable therapeutic option.

## Key Points

**Question:**

What are the mediators of repeated ketamine administration for chronic pain relief?

**Findings:**

In this cohort study of 329 patients with treatment-resistant pain, pretreatment depression was the only mediator of chronic pain relief with ketamine.

**Meaning:**

Depression as a mediator for refractory chronic pain relief provides radically new insights on how ketamine reduces pain primarily by dampening depression and reinforces the need for systematic depression assessment of patients with treatment-resistant pain.

## Teaser

This cohort study examines clinical pain trajectories with repeated ketamine administration to explore whether racemic ketamine dose, pretreatment depressive, or pretreatment anxiety symptoms mediate pain relief in patients with treatment-resistant chronic pain.

## Introduction

Drug management of chronic pain with antidepressants, antiepileptics, and opioids remains inconclusive, as 60% of patients show little improvement, experience adverse effects, and often require other treatment options.^1,2^ Ketamine, a nonselective *N*-methyl-d-aspartate receptor (NMDAR) antagonist with anesthetic properties, can relieve chronic pain.^3,4,5^ A recent clinical study^3^ in patients with refractory chronic pain identified distinct pain relief trajectories with variables that are associated with the response to a single dose of ketamine according to pain characteristics, level of anxiety, depression, or quality of life.

Comorbid pain and depression are common. In treatment-resistant depression, a number of studies^6,7,8,9^ have also reported ketamine to have rapid antidepressant and antisuicidal effects. Patients with chronic pain often experience depressive symptoms, as chronic pain-induced depression affects up to 85% of patients with chronic pain, depending on the clinical setting.^10^ Likewise, the prevalence of pain among patients with depression ranges from 43% to 80%.^11,12^ Both conditions are challenging and share clinical consequences of impaired function and decreased quality of life,^12,13^ which are accentuated when both are present in a patient. Depression may also be more difficult to alleviate in persons with concomitant pain.^12,14^

Pain and depression share common neurobiological elements in the central nervous system, at cerebral, brainstem, and spinal cord (descending inhibitory pathways) levels. This may explain concomitant decreased levels of pain and depression when ketamine is used.^15,16^ Few studies have evaluated this bilateral action of ketamine. Oral ketamine for 6 weeks showed an alleviation of depressive symptoms in chronic pain,^17^ and ketamine improved both depression and pain.^18^ A recent study^12^ evaluated the role of pretreatment pain symptoms in response to repeated ketamine infusions in individuals with depression and showed that patients with depressive symptoms and varying degrees of pain, especially severe pain, exhibited a significant and rapid improvement in depressive symptoms after 6 infusions of ketamine.

Conversely, there is limited information about the influence of pretreatment depressive symptoms on relief in patients with chronic pain treated with ketamine. To optimize the management of treatment-refractory chronic pain with ketamine, this 1-year study aimed to determine clinical pain trajectories with repeated ketamine administration, exploring whether racemic (R/S) ketamine dose and/or pretreatment depressive and/or anxiety symptoms mediate pain relief.

## Methods

### Study Design, Setting, and Population

This prospective, multicenter cohort study was conducted in 30 French pain clinics. Patients were followed up with telephone calls over 1 year by the Clinical Research Center and Clinical Investigation Center of Institut National de la Santé et de la Recherche Médicale (INSERM) 1405, Clermont-Ferrand University Hospital, Clermont-Ferrand, France. The study was approved by the National Ethics committee (Comité Consultatif sur le Traitement de l’Information en Matière de Recherche dans le Domaine de la Santé, Commission Nationale de L'informatique et des Libertés, and Comité de Protection des Personnes Sud-Est), was registered on ClinicalTrials.gov,^19^ and followed the Strengthening the Reporting of Observational Studies in Epidemiology (STROBE) reporting guideline for cohort studies. All participants provided written informed consent.

Male and female patients who were 18 years or older, had chronic pain for more than 6 months (peripheral or central neuropathic pain, fibromyalgia, complex regional pain syndrome, or other chronic pain), and required ketamine in their pain care pathway were eligible to participate in the pain clinic where they were usually treated. The clinician evaluated the eligibility criteria, explained the objectives of the study, gave an information and nonopposition form (approved by the National Ethics committee), and specified the patient could refuse to participate to the study.

This study included 585 patients. A previous publication^3^ focused on the outcomes of 256 patients who received only 1 ketamine administration. The present study focuses on the 329 patients who received more than 1 ketamine infusion.

### Drug Administration

Pain clinics followed their own R/S ketamine protocols, and these varied in terms of dosage, duration, frequency, and route of administration (eg, a single dose of 0.2 mg/kg over 40 minutes or 0.1 mg/kg/d once a week for 8 weeks, intravenous or subcutaneous). Cumulative dose in milligrams was used; for example, for a 70-kg person, a dose of 0.5 mg/kg/d once a month for 3 months was equal to a cumulative dose of 105 mg.

### Follow-up Procedure and Outcomes

After R/S ketamine administration, patients were called at 1 week and monthly for 1 year. Demographics, ketamine naivety, ketamine dosage, pain, comorbidities, questionnaires, concomitant analgesics, drug and nondrug treatments, and adverse effects were collected. The primary outcome, mean pain intensity, was assessed over 1 year with the Numerical Pain Rating Scale (NPRS) ranging from no pain (0) to maximal tolerable pain (10). Secondary outcomes included depression and anxiety scores measured using the Hospital Anxiety and Depression Scale (HADS),^20^ with scores ranging from 0 to 21 (≤7 indicates not pathological; 8-10, suspected case; ≥11, definite case); quality of life, using the 12-item Short Form Health Survey (SF-12)^21^ for mental and physical scores; cumulative ketamine dose (in milligrams); adverse effects; and concomitant treatments.

### Statistical Analysis

Data were analyzed from November 15 to December 31, 2022. Sample size estimation was determined sequentially according to rules of thumb for determining the minimum number of participants required for Cohen’s recommendations,^22^ with effect size (ES) bounds defined as small (ES = 0.2), medium (ES = 0.5), and large (ES = 0.8 [“grossly perceptible and therefore large”]). Therefore, with at least 320 patients evaluated at baseline and month 12, an ES greater than 0.3 (ie, 1-point difference for an SD of 3) can be highlighted for NPRS change, with a 2-sided type I error at 0.001 (correction due to multiple comparisons), a 90% statistical power, an intraindividual correlation coefficient (*r* value) of 0.5, and 15% lost to follow-up.

To analyze longitudinal data (NPRS and HADS), linear mixed models for repeated data were used, with time as a fixed effect and patient as a random effect, to account for between- and within-patient variability. Effect size and 95% CIs were calculated and interpreted according to Cohen’s recommendations.^22^

To identify distinctive trajectories of pain, semiparametric mixture models were used to model the association between pain and time for each trajectory, the shape of the trajectory, and the estimated proportion of the population belonging to each trajectory. The baseline characteristics of the patients were then compared according to the trajectories using the χ^2^ test or the Fisher exact test for categorical variables and analysis of variance or Kruskal-Wallis test for continuous variables.^23^

A mediation analysis was conducted to assess the respective contributions of the treatment dose and baseline depression to evolution of pain. A mediation proportion was estimated, indicating how much of the whole increment value provided by an independent variable can be explained by the indirect path in which changes in this independent variable drive a change in the mediator (retention rate) and changes in the mediator are associated with outcome. A multilevel mediation analysis was performed with sex and age being integrated. Results were expressed as mediation proportion and significance of the mediation analysis associations.

Unless otherwise indicated, data are expressed as mean (SD). Statistical analyses were performed using Stata software, version 15 (StataCorp LLC). All tests were 2 sided, with an α level set at 5%. The analyses were performed after the last-observation-carried-forward imputation method for missing data, for NPRS, HADS, and SF-12 (eMethods in Supplement 1).

## Results

### Patient Characteristics

**Figure 1.**
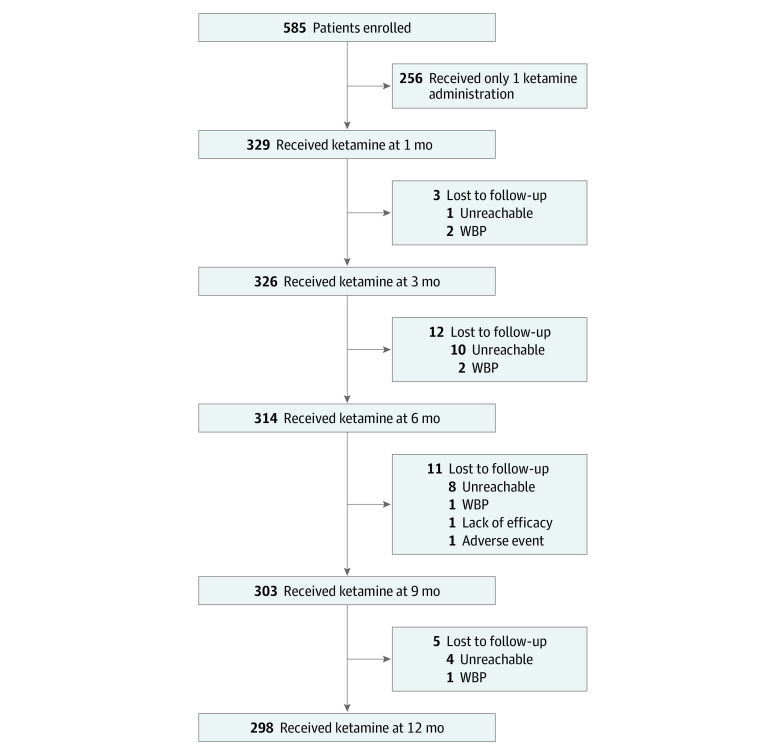
Study Flowchart WBP indicates withdrawal by patient.

Between July 7, 2016, and September 21, 2017, 329 patients (mean [SD] age. 51.4 [11.0] years; 249 women [75.7%] and 80 men [24.3%]) were included in the analysis. Participants received at least 2 ketamine administrations (Figure 1 and Table).

**Table.** Demographic and Clinical Characteristics of Patients at Baseline Before Ketamine Administration^a^

Characteristic	Total (n = 329)	Women (n = 249)	Men (n = 80)
Age, mean (SD), y	51.4 (11.0)	51.4 (10.8)	51.4 (11.6)
Patient history			
Neurological or psychological disorder	122 (37.1)	100 (40.2)	22 (27.5)
Gastrointestinal tract disease	89 (27.1)	73 (29.3)	16 (20.0)
Heart disease	59 (17.9)	42 (16.9)	17 (21.3)
Migraine	39 (11.9)	35 (14.1)	4 (5.0)
Pulmonary disorder	36 (10.9)	31 (12.4)	5 (6.3)
Thyroid disorder	28 (8.5)	24 (9.6)	4 (5.0)
Type 1 diabetes	23 (7.0)	19 (7.6)	4 (5.0)
Cancer	16 (4.9)	14 (5.6)	2 (2.5)
Ear, nose, and throat disorder	14 (4.3)	12 (4.8)	2 (2.5)
Psoriasis	9 (2.7)	6 (2.4)	3 (3.8)
Liver disease	6 (1.8)	6 (2.4)	0
Peripheral vessel disease	6 (1.8)	5 (2.0)	1 (1.3)
Osteoporosis	6 (1.8)	6 (2.4)	0
Prostate disorder	2 (0.6)	0	2 (2.5)
Chronic kidney disease	1 (0.3)	1 (0.4)	0
None	53 (16.1)	35 (14.1)	18 (22.5)
Pain-related			
Pain etiology			
Fibromyalgia	173 (52.6)	150 (60.2)	23 (28.8)
Peripheral neuropathic pain	101 (30.7)	62 (24.9)	39 (48.8)
Central neuropathic pain	18 (5.5)	8 (3.2)	10 (12.5)
Complex regional pain syndrome	21 (6.4)	17 (6.8)	4 (5.0)
Back pain, sciatica, cruralgia, neuralgia, pelvic pain, osteoarthritis	9 (2.7)	7 (2.8)	2 (2.5)
Rheumatoid arthritis, spondylitis	3 (0.9)	1 (0.4)	2 (2.5)
Headache	5 (1.5)	4 (1.6)	1 (1.3)
Other	3 (0.9)	1 (0.4)	2 (2.5)
DN4 (n = 135)^b^			
Mean (SD) score	5.6 (2.1)	5.6 (2.3)	5.7 (1.9)
≥4, No./total No. (%)	118/135 (87.4)	73/86 (84.9)	45/49 (91.8)
Mean pain intensity^c^			
Mean (SD) score	6.8 (1.8)	6.9 (1.8)	6.4 (1.7)
No./total No. (%) of patients			
<3	2/315 (0.6)	1/241 (0.4)	1/74 (1.4)
3-6	126/315 (40.0)	93/241 (38.6)	33/74 (44.6)
≥7	187/315 (59.4)	147/241 (61.0)	40/74 (54.1)
No. of pain paroxysms, median (IQR) (n = 206)	4 (3-8)	4 (2-8)	4 (3-10)
Maximal pain intensity^c^			
Mean (SD) score	8.4 (1.5)	8.4 (1.5)	8.3 (1.3)
No./total No. (%) of patients			
<3	1/313 (0.3)	1/238 (0.4)	0/75
3-6	28/313 (8.9)	20/238 (8.4)	8/75 (10.7)
≥7	284/313 (90.7)	217/238 (91.2)	67/75 (89.3)
Ketamine			
Ketamine naive	98 (29.8)	73 (29.3)	25 (31.3)
IV route	279 (84.8)	207 (83.1)	72 (90.0)
IV cumulative dose			
Median (IQR), mg	444 (280-666)	450 (280- 666)	444 (270- 718)
No./total No. (%) of patients			
≤280 mg	76/279 (27.2)	53/207 (25.6)	23/72 (31.9)
281-444 mg	64/279 (22.9)	50/207 (24.2)	14/72 (19.4)
445-666 mg	74/279 (26.5)	57/207 (27.5)	17/72 (23.6)
≥667 mg	65/279 (23.3)	47/207 (22.7)	18/72 (25.0)
IV duration			
Median (IQR), d	9 (6-12)	9 (6-10)	9 (6-12)]
No./total No. (%) of patients			
≤6 d	102/279 (36.6)	79/207 (38.2)	23/72 (31.9)
7-9 d	65/279 (23.3)	49/207 (23.7)	16/72 (22.2)
10-12 d	68/279 (24.4)	49/207 (23.7)	19/72 (26.4)
≥13 d	44/279 (15.8)	30/207 (14.5)	14/72 (19.4)
Emotional aspects			
HADS anxiety score^d^			
Mean (SD)	10.5 (4.3)	11.0 (4.3)	8.8 (4.0)
No./total No. (%) of patients			
≤7	91/313 (29.1)	60/236 (25.4)	31/77 (40.3)
8-10	65/313 (20.8)	44/236 (18.6)	21/77 (27.3)
≥11	157/313 (50.2)	132/236 (55.9)	25/77 (32.5)
HADS depression score^d^			
Mean (SD)	8.9 (4.2)	9.0 (4.3)	8.8 (3.9)
No./total No. (%) of patients			
≤7	118/312 (37.8)	88/236 (37.3)	30/76 (39.5)
8-10	75/312 (24.0)	56/236 (23.7)	19/76 (25.0)
≥11	119/312 (38.1)	92/236 (39.0)	27/76 (35.5)
Quality of life			
SF-12 physical score, mean (SD) (n = 294)	28.4 (8.0)	28.5 (8.1)	28.3 (8.0)
SF-12 mental score, mean (SD) (n = 294)	39.4 (10.8)	39.4 (10.5)	39.5 (11.7)
Concomitant drugs			
No. of treatments, mean (SD)	3.6 (1.9)	3.6 (1.9)	3.8 (2.2)
Paracetamol and/or NSAIDs	140 (42.6)	107 (43.0)	33 (41.3)
Step 2 opioids, nefopam^e^	180 (54.7)	138 (55.4)	42 (52.5)
Step 3 opioids^f^	52 (15.8)	33 (13.3)	19 (23.8)
Antidepressants	227 (69.0)	178 (71.5)	49 (61.3)
Antiepileptics	145 (44.1)	102 (41.0)	43 (53.8)
Adjuvants	66 (20.1)	50 (20.1)	16 (20.0)
Hypnotics and/or sedatives	60 (18.2)	44 (17.7)	16 (20.0)
Anxiolytics	106 (32.2)	84 (33.7)	22 (27.5)
Antipsychotics	20 (6.1)	14 (5.6)	6 (7.5)
Others	31 (9.4)	23 (9.2)	8 (10.0)
None	13 (4.0)	9 (3.6)	4 (5.0)

Abbreviations: DN4, Douleur Neuropathique 4 questionnaire; HADS, Hospital Anxiety and Depression Scale; IV, intravenous; NSAID, nonsteroidal anti-inflammatory drug; SF-12, 12-item Short Form Health Survey.

^a^ Unless otherwise indicated, data are expressed as No. (%) of patients. Missing data are not imputed.

^b^ Excludes fibromyalgia. Scores range from 0 to 10, with 4 or greater indicating neuropathic pain.

^c^ Scores range from no pain (0) to maximal tolerable pain (10).

^d^ Scores range from 0 to 21, with 7 or less indicating not pathological; 8 to 10, suspected case; and 11 or greater, definite case.

^e^ Includes dihydrocodeine, ibuprofen-codeine, paracetamol-codeine, paracetamol-opium, paracetamol-opium-caffeine, paracetamol-tramadol, tramadol, and tramadol-dexketoprofen.

^f^ Includes morphine, oxycodone, fentanyl, and buprenorphine. Multiple medications were frequent.

### Global Evolution Over 1 Year

Between baseline and month 12, the mean NPRS score decreased from 6.7 (1.8) to 5.6 (2.1), with a mean variation of −1.13 (2.22) (ES = −0.52 [95% CI, −0.62 to −0.41]; *P* < .001) (eFigure, A in Supplement 1). The mean HADS depression score decreased from 8.8 (4.2) to 7.5 (4.9) (ES = −0.44 [95% CI, −0.54 to −0.33]; *P* < .001), with 123 definite cases (37.4%; score ≥11) at baseline and 94 (28.6%) at month 12 (eFigure, B2 in Supplement 1). The mean HADS anxiety score decreased from 10.4 (4.3) to 8.7 (4.6) (ES = −0.58 [95% CI, −0.69 to −0.47]; *P* < .001), with 163 definite cases (49.5%; score ≥11) at baseline and 109 (33.1%) at month 12 (eFigure, B1 in Supplement 1). At baseline, a combination of definite depression (score ≥11) and anxiety (score ≥11) affected 92 patients (28.0%), and 60 (18.2%) also had an NPRS score of greater than 7.

**Figure 2.**
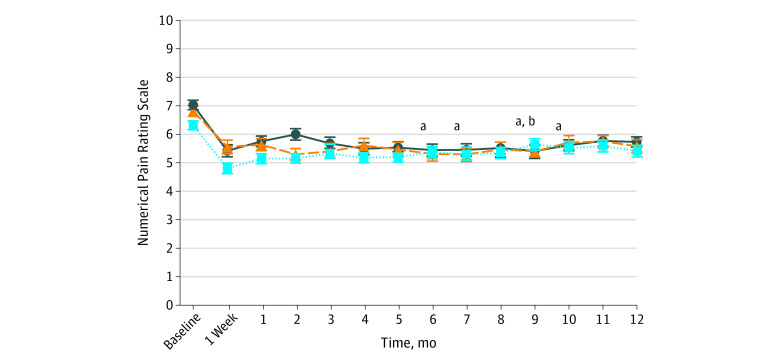
Pain Intensity Depending on Baseline Depression Status Data are presented as mean (SEM). Missing data for mean pain intensity are imputed with the last-observation-carried-forward method. The dark blue line represents patients with a Hospital Anxiety and Depression Scale (HADS) score ≥11 (definite case) (n = 123); orange dashed line, patients with a HADS score of 8-10 (suspected case) (n = 77); and light blue dotted line, patients with a HADS score ≤7 (not pathological) (n = 129). ^a^*P* < .05 between time (compared with baseline) and baseline depression status (Hospital Anxiety and Depression Scale scores ≤7 [not pathological] vs ≥11 [definite case]). ^b^*P* < .05 between time (vs baseline) and baseline depression status (Hospital Anxiety and Depression Scale scores ≤7 [not pathological] vs 8-10 [suspected case]).

Evolution of pain over time according to baseline depression showed an overall decrease in pain for all patients, the benefits of repeated administrations of ketamine being observed in patients both with and without depression disorders. According to baseline depression level, there was a significant difference between depressive (HADS score >7) and nondepressive (HADS score ≤7) scores in pain diminution (regression coefficient, −0.04 [95% CI, −0.06 to −0.01]; omnibus *P* = .002 for interaction of time × baseline depression status ≤7 or >7) (Figure 2).

Evolution of pain over time according to anxiety at baseline showed an overall decrease in pain for all patients, the benefits of repeated infusions of ketamine being observed in patients both with and without anxiety disorders. There was no difference in pain evolution according to baseline anxiety level (regression coefficient, −0.01 [95% CI, −0.03 to 0.02]; omnibus *P* = .56 for interaction of time × baseline anxiety status ≤7 or >7).

The mean mental health dimension scores of SF-12 increased between baseline and month 12, from 39.7 (10.9) to 42.2 (11.1) (*P* < .001). Mean physical health dimension scores increased from 28.5 (7.9) to 29.5 (9.2) (*P* = .02) (eFigure, C in Supplement 1).

At 1 week after ketamine administration, 123 of 297 patients (41.4%) experienced at least 1 adverse effect; at 1 month, 68 of 289 (23.5%); and for the rest of the year, between 28 of 220 (12.7%) and 59 of 277 (21.3%). The main adverse effects were fatigue (184 of 645 [28.5%] of all adverse effects collected), nausea (118 of 645 [18.3%]), headache (115 of 645 [17.8%]), and drowsiness (59 of 645 [9.1%]) (eFigure, D in Supplement 1).

Overall, concomitant treatments were not changed between baseline and month 12, except for acetaminophen (paracetamol) and/or nonsteroidal anti-inflammatory drugs (140 of 329 [42.6%] and 155 of 291 [53.3%], respectively; *P* < .001), step 3 opioids (52 of 329 [15.8%] and 54 of 291 [18.6%], respectively; *P* = .04), and antiepileptics (145 of 329 [44.1%] and 123 of 291 [42.3%], respectively; *P* = .01). About half of patients had kinesiotherapy or mild physical activity during follow-up.

### Pain Trajectories

**Figure 3.**
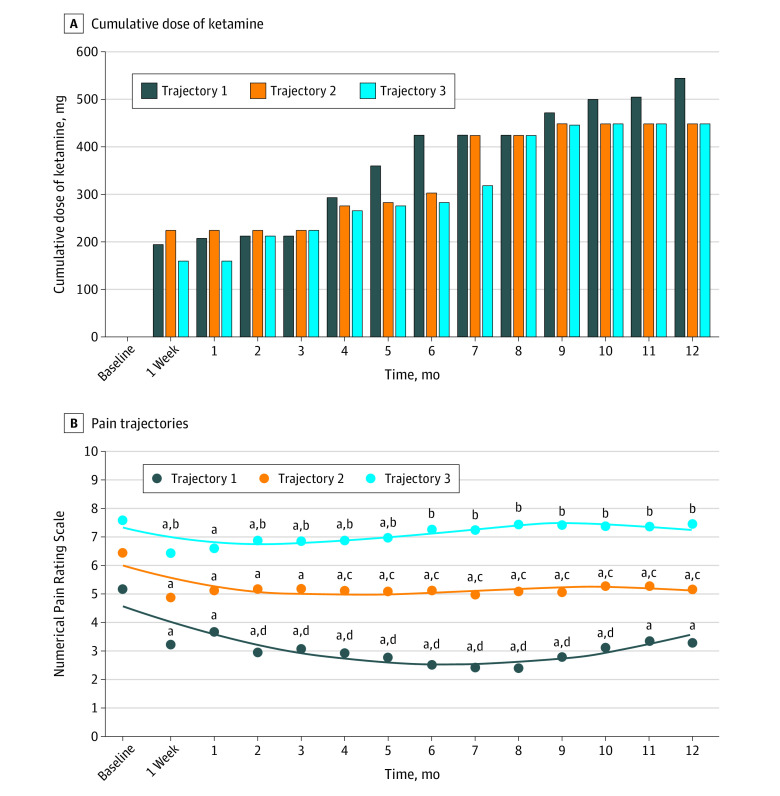
One-Year Pain Trajectories in Patients After Intravenous Ketamine Administration (n = 279) A, Median cumulative dose of ketamine in each trajectory and at each time point. B, Pain trajectories obtained using semiparametric mixture models. ^a^*P* < .05 vs baseline (intragroup analysis). ^b^*P* < .05 between time (vs baseline) pain trajectories (trajectory 3 vs trajectory 1). ^c^*P* < .05 between time (vs baseline) and pain trajectories (trajectory 2 vs trajectory 3). ^d^*P* < .05 between time (vs baseline) and pain trajectories (trajectory 1 vs trajectory 2).

Three pain trajectories (T1, T2, and T3) were identified in 279 patients who received intravenous ketamine. Mean NPRS score at baseline was 5.3 (1.7) in T1 (lesser pain; 52 [18.6%]), 6.5 (1.5) in T2 (moderate pain; 134 [48.0%]), and 7.8 (1.5) in T3 (severe pain; 93 [33.3%]) (Figure 3). There was a significant difference between pain trajectories about pain etiology, especially fibromyalgia (15 of 52 [28.8%] in T1, 83 of 134 [61.9%] in T2, and 45 of 93 [48.4%] in T3; *P* < .001). Patients with neuropathic pain (18 of 52 [34.6%]) in T1 have alleviation of pain with NPRS scores less than 3 from month 3.

Concerning the HADS, mean baseline depression scores increased from T1 to T3 (7.9 [4.1] in T1, 8.7 [4.0] in T2, and 9.4 [4.2] in T3; *P* = .09) and mean baseline anxiety scores increased significantly (9.6 [3.9] in T1, 10.1 [4.4] in T2, and 11.5 [4.1] in T3; *P* = .009). Scores of mental and physical health dimensions of SF-12 decreased from T1 to T3 (eTable 1 in Supplement 1). Cumulative ketamine dose was not associated with pain trajectories, whatever the time of evaluation. There was a significant interaction between pain trajectories and time. The time point at which the variation from baseline started to be significantly different between the trajectories was at month 2 between T1 and T3 (regression coefficient, 1.51 [95% CI, 0.78-2.23]; *P* < .001), at month 2 (until month 10) between T1 and T2 (regression coefficient, 1.03 [95% CI, 0.34-1.71]; *P* = .003), and at month 4 between T2 and T3 (regression coefficient, 0.59 [95% CI, 0.03-1.16]; *P* = .04) (Figure 3). Finally, the baseline number of ketamine-naive patients, number of treatments, and the follow-up concomitant treatments did not differ between pain trajectories (eTable 2 in Supplement 1).

### Mediation

**Figure 4.**
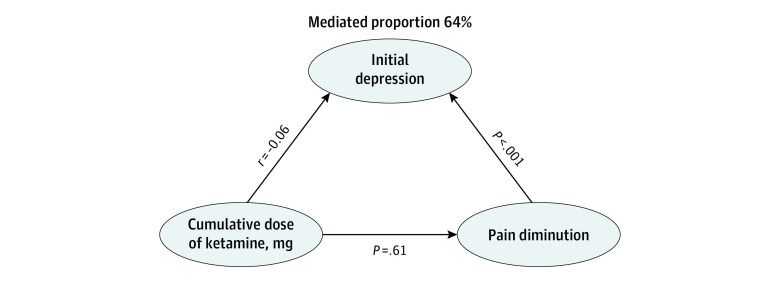
Multilevel Mediation Method

The multilevel mediation model method provided information from several path models. In path 1, ketamine dose was not associated with pain diminution (*r* = 0.01; *P* = .61). In path 2, ketamine dose was not correlated with depression (*r* = −0.06; *P* = .32). In path 3, multivariate analysis showed depression was associated with pain diminution (regression coefficient, 0.03 [95% CI, 0.01-0.04]; *P* < .001), whereas ketamine dose was not (regression coefficient, 0.00 [95% CI, −0.01 to 0.01]; *P* = .67). In other words, proportion of reduction of pain mediated by ketamine dose was 0% and 64.6% for baseline depression (Figure 4).

## Discussion

In this 1-year clinical cohort study, repeated R/S ketamine treatment in chronic refractory pain was associated with pain relief for all patients with or without concomitant depression and/or anxiety. Ketamine was immediately associated with a diminution of pain, confirming results of some of the literature^3,24^ and of a previous clinical study,^3^ where a single subanesthetic dose of ketamine induced rapid and sustained pain relief. Likewise, ketamine was associated with rapid and sustained improvement of depression, as reported previously.^25^ Concerning adverse effects, these were in the usual range described for ketamine use, and ketamine was overall well tolerated and associated with significantly improved quality of life.

Repetition of ketamine administration does not, however, provide more analgesia than the immediate relief at 1 week, suggesting a threshold beyond which ketamine cannot diminish pain any further and that the analgesic effects of ketamine occur mainly at the start of the treatment. Cumulative ketamine dose was not associated with pain relief and not involved in the mediation model, underlining that trajectories and profiles of responders are independent from the dose. However, with repeated ketamine administration, the percentage of patients in the trajectory with most severe pain was only 33.3% compared with the 48.0% we observed with a single dose,^3^ suggesting that ketamine may globally have an analgesic effect on more patients with severe pain. The 3 pain trajectories allow the identification of different profiles that are rather similar to those with a single ketamine administration.^3^ Patients with neuropathic pain (18 of 52 [34.6%]) in T1 have alleviation of pain with NPRS scores less than 3 from month 3, while diminution of pain in patients with fibromyalgia, who account for 83 of 134 (61.9%) in T2 and 45 of 93 (48.4%) in T3, occurs only briefly.

The total rate of depression at baseline in our study, 75 of 312 suspected cases (24.0%) and 119 of 312 definite cases (38.1%), is high (higher than what has been described in some studies^26^), and 60 of 329 patients (18.2%), the most vulnerable of the cohort, combined highest depression and/or anxiety severity and highest pretreatment pain scores. We observed that patients with pretreatment depression (score >7) displayed more robust symptomatic pain improvement, with the amplitude of pain decrease associated with the severity of baseline depression. This mediation of depression in pain relief was not associated with the pain trajectories and occurred whatever the pain trajectory. Anxiety, however, does not present the same correlation as depression; this is in accordance with some published research, but ketamine effect on anxiety is still controversial.^27^

The link between depression alleviation (especially for depression with suicidal ideations)^6,28^ and pain relief^5^ with ketamine treatment is complex.^24,29^ Comorbid pain and depression are frequent, with a bidirectional interaction,^26,30^ but a recent activation likelihood estimation meta-analysis^31^ stresses that the direction of comorbidity (ie, pain with depression vs depression with pain) is rarely addressed and may concern different cerebral areas and neurobiology mechanisms. The analysis indicated that pain with concomitant depression was associated with the right amygdala, while depression with concomitant pain was related primarily to the left dorsolateral prefrontal cortex. The amygdala, its adjacent limbic structures such as the hippocampus, and the connectivity between the medial prefrontal cortex and these limbic structures are implicated in chronic pain. As depression appears in our study as the main mediator (64.6% of the mediation) of the analgesic effect of ketamine, this suggests that ketamine mode of action might be primarily directed toward the prefrontal cortex and probably involves and modulates the frontostriatal circuitry thereafter.^32^

In effect, there are overlaps in the neurobiological and clinical aspects of these comorbidities, with the involvement of NMDAR, inflammation, and psychological elements. Ketamine mechanism of action involves numerous receptors, metabolites, and targets common to pain and depression,^33,34^ with a number of downstream mechanisms that will regulate synaptic plasticity. Inhibition of NMDAR is pivotal for antidepressant and pain alleviation effects, with direct synaptic, extrasynaptic, and γ-aminobutyric acid–stimulating interneuron NMDAR inhibition. Ketamine may also modulate inflammation as shown when markers of inflammation and cytokines are elevated.^18,35^ Another point concerns cognition, which is impaired in pain^36^ and in depression.^37,38,39^ Ketamine has an action at a cognitive level, with belief updating becoming more optimistically biased rapidly after the first ketamine infusion.^40^

### Limitations

This study has some limitations. Depression was screened with HADS, as is commonly done in patients with chronic pain, and not with Montgomery-Åsberg Depression Rating Scale, which is used for depression evaluation.^7^ As a clinical study, there is no placebo group, and the placebo effect of ketamine that is not negligible^24^ has not been evaluated. Subjective psychopathology and neurocognitive performance after starting the infusion were not measured. It would be worth adding them in future trials. Furthermore, R/S ketamine was used, and it would be interesting to compare with other enantiomers, as differences have been described.^41^

## Conclusion

The findings of this cohort study suggest that depression (and not ketamine dose or anxiety) may be the mediator of the diminution of pain with repeated administrations of ketamine. This finding provides new insight into how ketamine may reduce pain primarily by dampening depression. This reinforces the need for systematic holistic assessment of patients with chronic pain to diagnose severe depressive symptoms where ketamine would be a very valuable therapeutic option. Further research is needed on the mechanistic, biological, and cognitive properties of ketamine in comorbid pain and depression.

## Notes

Supplement 1. **eMethods.** Statistical Analysis **eFigure.** One-Year Evolution of Numerical Pain Rating Scale **eTable 1.** Initial Baseline Characteristics of Patients According to Pain Trajectories After Intravenous Ketamine (n = 279) **eTable 2.** Concomitant Treatments Depending on Pain Trajectories During the Year of Follow-up After Intravenous Ketamine (n = 279)

Supplement 2.
Data Sharing Statement
