# Supplementary material for: Assessment of Initial Depressive State and Pain Relief With Ketamine in Patients With Chronic Refractory Pain
Source: JAMA Netw Open. 2023 May 19;6(5):e2314406. doi: 10.1001/jamanetworkopen.2023.14406 (PMC10199354; doi:10.1001/jamanetworkopen.2023.14406)
Supplement: Supplement 1. — eMethods. Statistical Analysis eFigure. One-Year Evolution of Numerical Pain Rating Scale eTable 1. Initial Baseline Characteristics of Patients According to Pain Trajectories After Intravenous Ketamine (n = 279) eTable 2. Concomitant Treatments Depending on Pain Trajectories During the Year of Follow-up After Intravenous Ketamine (n = 279) [file jamanetwopen-e2314406-s001.pdf]

Supplementary Online Content

Voute M, Lambert C, Pereira B, Pickering G. Assessment of initial depressive state and pain relief with ketamine in patients with chronic refractory pain. *JAMA Netw Open*. 2023;6(5):e2314406. doi:10.1001/jamanetworkopen.2023.14406

eMethods. Statistical Analysis

eFigure. One-Year Evolution of Numerical Pain Rating Scale

eTable 1. Initial Baseline Characteristics of Patients According to Pain Trajectories After Intravenous Ketamine (n = 279)

eTable 2. Concomitant Treatments Depending on Pain Trajectories During the Year of Follow-up After Intravenous Ketamine (n = 279)

This supplementary material has been provided by the authors to give readers additional information about their work.

© 2023 Voute M et al. *JAMA Network Open*.

eMethods. Statistical Analysis

Sample size estimation was determined sequentially according to rules-of-thumb for determining the minimum number of subjects required to Cohen's recommendations[21] who has defined effect size (ES) bounds as: small (ES=0.2), medium (ES=0.5) and large (ES=0.8, "grossly perceptible and therefore large"). So, with at least 320 patients evaluated at baseline and month 12, an ES greater than 0.3 (i.e. 1-point difference for a standard deviation of 3) can be highlighted for numerical pain rating scale change, with a two-sided type I error at 0.001 (correction due to multiple comparisons), a 90% statistical power, an intra-individual correlation coefficients equals 0.5 and 15% of lost to follow-up.

The categorical data are expressed as numbers and associated percentages, and the continuous data as mean ± standard-deviation or median [25th; 75th percentiles], based on the statistical distribution.

To analyze longitudinal data (NPRS and HADS), linear mixed models for repeated data were performed, with time as fixed effect and patient as random-effect, to take into account between and within patient variability. ES and 95% confidence interval (95%CI) were calculated between baseline and month 12, and interpreted according to Cohen's recommendations aforementioned. The evolution of pain over time according to depression and anxiety levels at baseline was studied with linear mixed models with the following fixed effects: depression or anxiety levels (≤7, 8 to 10, ≥11), time of measurement (baseline, week 1, month 1 to month 12) and their interaction "HADS levels x time" (P-values denoted Pi). The normality of residuals was studied using the Shapiro-Wilk test. When appropriate, a logarithmic transformation was proposed to achieve the normality of the dependent outcome.

To identify distinctive trajectories of pain, semi-parametric mixture models (group-based trajectory model) were carried out to model the relationship between pain and time, for each trajectory, the shape of the trajectory and the estimated proportion of the population belonging to each trajectory. These probabilities are called the posterior probability of group membership. To create the profiles, individuals were assigned to the trajectory group to which they most likely belonged based on their measured history of pain. Groupings may identify distinct subpopulations. The analysis provides a formal way to determine the best-fit number of trajectories and a precision estimate of group membership allocation which can be expressed using observed probabilities and posteriori probabilities. Nagin lays out several statistically oriented criteria for assessing model adequacy[22]. These include: (a) obtaining for each trajectory group a close correspondence between the estimated probability of group

© 2023 Voute M et al. *JAMA Network Open*.

membership and the proportion assigned to that group based on the posterior probability of group membership, (b) ensuring that the average of the posterior probabilities of group membership for individuals assigned to each group exceeds a minimum threshold of 0.7, (c) establishing that the odds of correct classification based on the posterior probabilities of group membership exceed a minimum threshold of 5, and (d) observing reasonably tight confidence intervals around estimated group membership probabilities. Furthermore, the best-fitting model was selected according to the Bayesian Information Criterion. Then, the baseline characteristics of the patients were compared according to the trajectories using the Chi-squared test or the Fisher's exact test for categorical variables, and ANOVA or Kruskal-Wallis test for continuous variables.

A mediation analysis was conducted to assess the respective contributions of the treatment dose and baseline depression on evolution of pain. A mediation proportion was estimated, indicating how much of the whole increment value provided by an independent variable can be explained by the indirect path in which changes in this independent variable drives a change in the mediator (retention rate), and changes in the mediator then affect outcome. A multilevel mediation analysis was performed with other sex and age being integrated. Results were expressed as mediation proportion and significance of the mediation analysis associations.

Statistical analyses were performed using Stata software (version 15; StataCorp, College Station, Texas, USA). All tests were two-sided, with an alpha level set at 5%. P-values are denoted "P", except for interaction terms ("Pi").The analyses were performed after the last observation carried forward imputation method for missing data, for NPRS, HADS and SF-12.

© 2023 Voute M et al. *JAMA Network Open*.

eFigure. One-Year Evolution of Numerical Pain Rating Scale

(A), anxiety (B1), depression (B2), quality of life (C) and adverse effects (D). Data are presented as mean ± standard deviation in Figures A, B1, B2 and C, and as percentages in figure D. Missing data for numerical pain rating scale, anxiety, depression and quality of life are imputed with the last observation carried forward imputation method. HADS, Hospital Anxiety and Depression Scale; M, months; SF12-12 item Short Form health survey. * Significant difference compared to baseline (* P<.05, ** P<.01, *** P<.001).

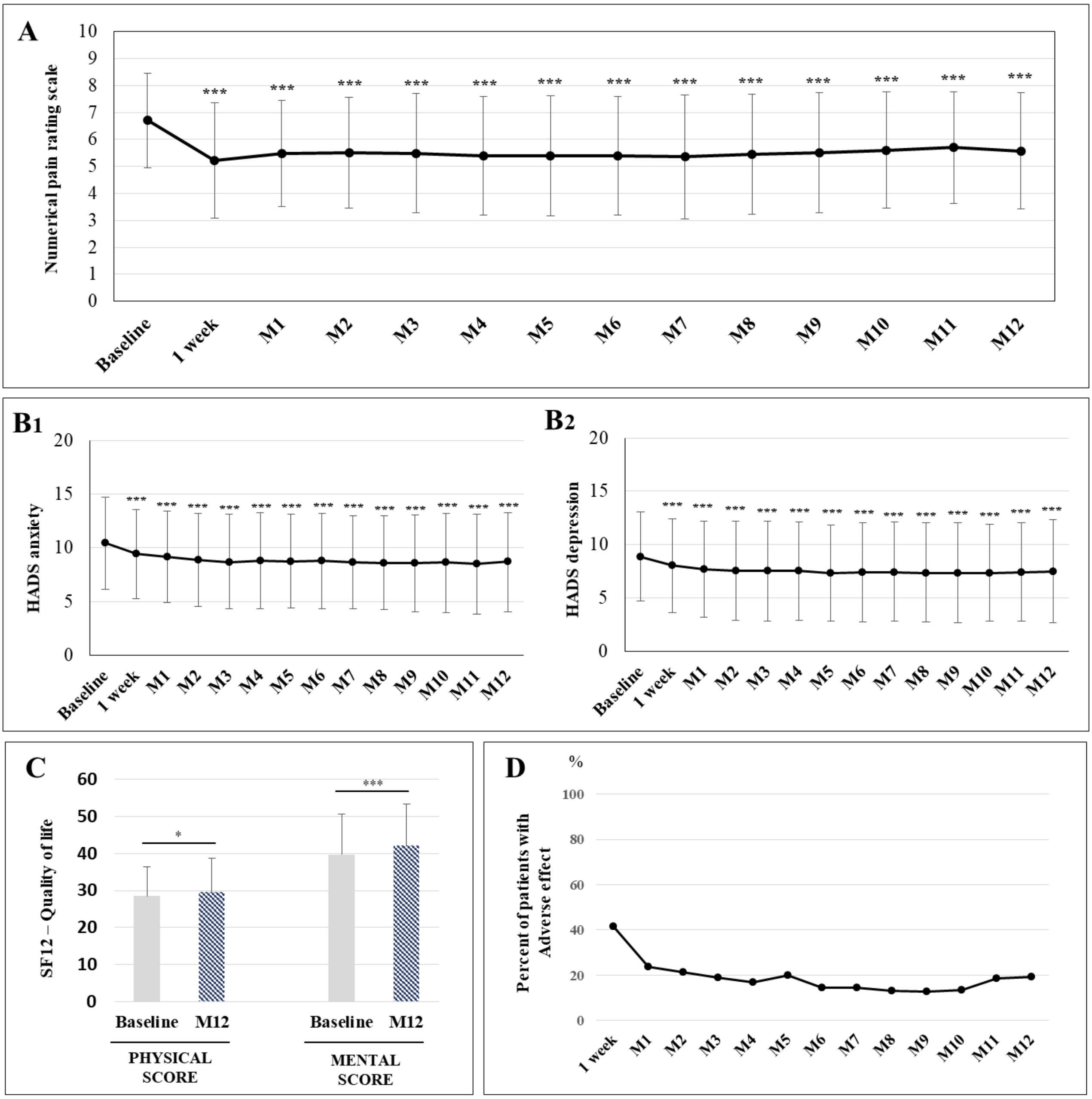

eTable 1. Initial Baseline Characteristics of Patients According to Pain Trajectories After Intravenous Ketamine (n = 279)

Data are presented as number of patients (percentages), mean ± standard deviation, or median [25th; 75th percentiles]. In this table, missing data for average pain intensity, HADS and SF-12 are imputed with the last observation carried forward imputation method. DN4, Douleur Neuropathique 4; HADS, Hospital Anxiety and Depression Scale; NSAIDs, Non-Steroidal Anti-Inflammatory Drugs; SF-12, 12-item Short Form health survey; WHO, World Health Organization. [a] DN4: except for fibromyalgia. [b] Step 2 (WHO) opioids: dihydrocodeine; ibuprofene-codeine; paracetamol-codeine; paracetamol-opium; paracetamol-opium-cafeine; paracetamol-tramadol; tramadol; tramadol-deketopofene. [c] Step 3 (WHO) opioids: morphine; oxycodone; fentanyl; buprenorphine.

	**Trajectory 1 (n=52)**	**Trajectory 2 (n=134)**	**Trajectory 3 (n=93)**	***P***
**DEMOGRAPHICS**				
Age (years)	52.8±9.2	50.9±11.5	51.4±11.2	.58
Female sex	36 (69.2)	97 (72.4)	74 (79.6)	.32
**PAIN-RELATED**				
Pain etiology				
Fibromyalgia	15 (28.8)	83 (61.9)	45 (48.4)	<.001
Peripheral neuropathic pain	18 (34.6)	33 (24.6)	34 (36.6)	.12
Central neuropathic pain	6 (11.5)	5 (3.7)	4 (4.3)	.12
Complex regional pain syndrome	6 (11.5)	6 (4.5)	8 (8.6)	.19
Back pain, sciatica, cruralgia, neuralgia, pelvic pain, osteoarthritis	3 (5.8)	4 (3.0)	1 (1.1)	.25
Rheumatoid arthritis, spondylitis	2 (3.8)	1 (0.7)	0 (0)	.13
Headache	1 (1.9)	2 (1.5)	2 (2.2)	>.99
Others	2 (3.8)	0 (0)	0 (0)	.03
DN4[a] (0-10) *(n=116)*	5.8±2.0	5.7±2.1	5.5±1.8	.83
DN4[a] ≥4	28/33 (84.8)	36/40 (90.0)	39/43 (90.7)	.76
Average pain intensity (0-10)	5.3±1.7	6.5±1.5	7.8±1.5	<.001
<3	1 (1.9)	1 (0.7)	0 (0)	
3 to 6	38 (73.1)	62 (46.3)	12 (12.9)	<.001
≥7	13 (25.0)	71 (53.0)	81 (87.1)	
Number of pain paroxysms *(n=182)*	4 [2; 6]	5 [3; 10]	4 [2; 9]	.08
Maximal pain intensity (0-10) *(n=265)*	7.1±1.9	8.3±1.3	9.1±0.9	<.001
<3	0/49 (0)	0/128 (0)	0/88 (0)	
3 to 6	16/49 (32.7)	9/128 (7.0)	1/88 (1.1)	<.001
≥7	33/49 (67.3)	119/128 (93.0)	87/88 (98.9)	
**KETAMINE**				

© 2023 Voute M et al. *JAMA Network Open*.

	Trajectory 1 (n=52)	Trajectory 2 (n=134)	Trajectory 3 (n=93)	*P*
Ketamine naive	13 (25.0)	36 (26.9)	36 (38.7)	.10
IV Cumulative dose (mg)	539 [420; 735]	444 [300; 666]	444 [210; 666]	.23
IV Cumulative dose >444 mg	34 (65.4)	63 (47.0)	42 (45.2)	.04
Duration (days)	10 [6; 12]	9 [6; 10]	8 [6; 11]	.21
Duration >9 days	26 (50.0)	51 (38.1)	35 (37.6)	.27
**EMOTIONAL ASPECTS**				
HADS, anxiety score (0-21)	9.6±3.9	10.1±4.4	11.5±4.1	.009
≤7	19 (36.5)	43 (32.1)	20 (21.5)	
8 to 10	13 (25.0)	29 (21.6)	14 (15.1)	.04
≥11	20 (38.5)	62 (46.3)	59 (63.4)	
HADS, depression score (0-21)	7.9±4.1	8.7±4.0	9.4±4.2	.09
≤7	26 (50.0)	53 (39.5)	33 (35.5)	
8 to 10	11 (21.2)	34 (25.4)	20 (21.5)	.38
≥11	15 (28.8)	47 (35.1)	40 (43.0)	
**QUALITY OF LIFE**				
SF-12, physical score	30.7±9.1	29.0±8.4	26.9±6.0	.03
SF-12, mental score	43.5±10.4	39.4±10.7	37.7±10.8	.004
**CONCOMITANT DRUGS**				
Number of treatments	3.5±1.9	3.3±1.9	3.9±2.1	.13
Paracetamol/NSAIDs	22 (42.3)	64 (47.8)	34 (36.6)	.24
Step 2 opioids[b], nefopam	22 (42.3)	75 (56.0)	53 (57.0)	.18
Step 3 opioids[c]	8 (15.4)	17 (12.7)	17 (18.3)	.51
Antidepressants	33 (63.5)	89 (66.4)	67 (72.0)	.51
Antiepileptics	26 (50.0)	45 (33.6)	44 (47.3)	.04
Adjuvants	11 (21.2)	24 (17.9)	21 (22.6)	.67
Hypnotics/sedatives	10 (19.2)	19 (14.2)	18 (19.4)	.52
Anxiolytics	14 (26.9)	36 (26.9)	37 (39.8)	.09
Antipsychotics	5 (9.6)	3 (2.2)	9 (9.7)	.02
Others	6 (11.5)	14 (10.4)	10 (10.8)	.98
None	1 (1.9)	5 (3.7)	6 (6.5)	.50

© 2023 Voute M et al. *JAMA Network Open*.

eTable 2. Concomitant Treatments Depending on Pain Trajectories During the Year of Follow-up After Intravenous Ketamine (n = 279)

Data are presented as percentages. In this table, missing data are not imputed. M, months; NSAIDs, Non-Steroidal Anti-Inflammatory Drugs; T, trajectories; WHO, World Health Organization. [a]Step 2 (WHO) opioids: dihydrocodeine; ibuprofene-codeine; paracetamol-codeine; paracetamol-opium; paracetamol-opium-cafeine; paracetamol-tramadol; tramadol; tramadol-deketopofene. [b]Step 3 (WHO) opioids: morphine; oxycodone; fentanyl; buprenorphine. Antidepressants : tricyclics, Selective Serotonin Reuptake Inhibitors, Serotonin-norepinephrine reuptake inhibitors ... Antiepileptics : pregabalin, gabapentin, carbamazepine… Adjuvants : triptans, lidocaïn5% plaster; corticosteroids…Hypnotics/sedatives/anxiolytics :benzodiazepines, buspirone... Antipsychotics : olanzapine, risperidone… * Significant difference (omnibus *P*<.05) between trajectories.

	**Trajectories**	**Baseline**	**1 week**	**M1**	**M2**	**M3**	**M4**	**M5**	**M6**	**M7**	**M8**	**M9**	**M10**	**M11**	**M12**
**Paracetamol/NSAIDs**	T1	42.3	46.7	47.9	48.0	48.1	50.0	48.1	48.0	48.0	48.9	49.0	50.0	54.5	50.0
	T2	47.8	50.0	51.5	53.0	56.5	54.9	55.7	56.3	55.2	56.0	57.0	58.5	56.5	58.8
	T3	36.6	39.8	39.3	43.0	45.1	43.8	46.7	47.2	47.7	47.1	47.7	48.8	46.8	47.6
**Step 2 opioids[a], nefopam**	T1	42.3	40.0	37.5	32.0*	34.6*	34.6*	30.8*	30.0*	30.0*	32.7*	32.7*	33.3*	34.1*	35.4
	T2	56.0	57.1	55.4	58.2*	57.3*	57.1*	56.5*	56.3*	55.2*	54.4*	55.4*	54.2*	52.2*	50.9
	T3	57.0	56.6	57.3	55.9*	57.1*	57.3*	55.6*	57.3*	58.1*	58.6*	58.1*	58.1*	58.2*	57.1
**Step 3 opioids[b]**	T1	15.4	17.8	18.8	16.0	17.3	17.3	17.3	18.0	18.0	18.4	18.4	18.8	16.0	18.8
	T2	12.7	11.1	12.3	11.2	12.2	11.3	10.0	11.0	11.2	11.2	10.7	11.9	10.4	12.3
	T3	18.3	20.5	21.4	21.5	22.0	20.2	20.0	21.4	20.9	21.8	20.9	22.1	22.8	23.8
**Antidepressants**	T1	63.5	60.0	58.3	58.0	57.7	57.7	57.7	56.0	56.0	55.1	57.1	58.3	59.1	58.3
	T2	66.4	69.8	66.9	67.2	67.1	69.2	66.4	66.4	65.6	66.4	66.1	65.3	65.2	64.0
	T3	72.0	71.1	74.2	73.1	72.5	71.9	73.3	74.2	74.4	73.6	73.3	73.3	76.0	72.6
**Antiepileptics**	T1	50.0*	51.1	47.9	48.0	48.1	48.1	48.1	50.0	50.0	49.0	51.1	52.1	52.3	45.8
	T2	33.6*	35.7	37.7	35.1	35.1	36.8	36.6	36.7	36.0	36.8	35.5	35.6	34.8	35.1
	T3	47.3*	47.0	47.2	49.5	50.0	48.3	46.6	44.9	44.2	44.8	45.4	45.6	44.3	45.3
**Adjuvants**	T1	21.2	22.2	20.1	20.0	19.2	19.2	19.2	18.0	20.0	18.4	18.4	18.8	20.5	18.8
	T2	17.9	20.6	22.3	23.1	20.6	21.8	22.9	22.7	21.6	21.6	22.3	22.0	20.9	21.9
	T3	22.6	22.9	24.8	23.7	23.1	22.5	23.3	23.6	24.4	24.1	23.3	24.4	22.8	21.4

© 2023 Voute M et al. *JAMA Network Open*.

	Trajectories	Baseline	1 week	M1	M2	M3	M4	M5	M6	M7	M8	M9	M10	M11	M12
**Hypnotics/sedatives**	T1	19.2	17.8	16.7	18.0	19.2	19.2	19.2	20.0	20.0	20.4	20.4	20.8	18.2	18.8
	T2	14.2	14.3	14.7	15.7	14.5	14.3	13.7	12.5	12.8	12.0	12.4	13.6	14.8	17.5
	T3	19.4	20.5	20.2	19.4	19.8	21.4	21.1	21.4	22.1	21.8	22.1	22.1	21.5	22.6
**Anxiolytics**	T1	26.9	24.4	22.9*	26.0*	23.1*	23.1*	23.1*	22.0*	22.0*	22.5*	22.5*	25.0*	25.0*	25.0*
	T2	26.9	28.6	26.2*	27.6*	29.0*	28.6*	27.5*	26.6*	24.0*	24.0*	22.3*	22.9*	22.6*	19.3*
	T3	39.8	36.1	40.5*	40.9*	41.8*	41.6*	42.2*	42.7*	44.2*	43.7*	44.2*	46.5*	48.1*	45.2*
**Antipsychotics**	T1	9.6*	11.1*	10.4*	8.0*	7.7*	7.7*	7.7*	8.0*	8.0*	8.2*	8.2*	8.3*	9.1*	8.3*
	T2	2.2*	2.4*	1.5*	2.2*	2.3*	2.3*	2.3*	1.6*	1.6*	1.6*	1.7*	1.7*	1.7*	1.8*
	T3	9.7*	10.8*	10.1*	9.7*	8.8*	7.9*	8.9*	9.0*	9.3*	9.2*	9.3*	9.3*	8.9*	9.5*

© 2023 Voute M et al. *JAMA Network Open*.
