# Supplementary material for: Assessment of Initial Depressive State and Pain Relief With Ketamine in Patients With Chronic Refractory Pain
Source: JAMA Netw Open. 2023 May 19;6(5):e2314406. doi: 10.1001/jamanetworkopen.2023.14406 (PMC10199354; doi:10.1001/jamanetworkopen.2023.14406)
Supplement: Supplement 2. — Data Sharing Statement [file jamanetwopen-e2314406-s002.pdf]

Data Sharing Statement

Voute. Assessment of Initial Depressive State and Pain Relief With Ketamine in Patients With Chronic Refractory Pain. *JAMA Netw Open*. Published May 19, 2023.
doi:10.1001/jamanetworkopen.2023.14406

Data
Data available: No
